# Supraseptal groove fold-in flap for the reconstruction of middle vault in primary rhinoplasty

**DOI:** 10.1016/j.jpra.2025.06.005

**Published:** 2025-06-18

**Authors:** Yakup Karabağlı, Can Ekinci, Sabri Karabağlı, Gülsen Özşahin

**Affiliations:** a Department of Plastic, Reconstructive and Aesthetic Surgery, Eskişehir Osmangazi University School of Medicine, Prof. Dr. Nabi Avcı Street, Eskişehir, Turkey; b Department of Plastic, Reconstructive and Aesthetic Surgery, Zonguldak Bülent Ecevit University School of Medicine, Esenköy Street, Zonguldak, Turkey

**Keywords:** Cartilage, Nasal septum, Nose, Reconstruction, Rhinoplasty

## Abstract

**Background:**

During primary rhinoplasty, dorsal hump reduction almost always impairs the middle vault and if not reconstructed nasal obstruction is likely to occur.

**Objective:**

The aim of this study is to suggest a novel technique that can utilize supraseptal groove as fold-in flap to widen and support the middle vault for better functional results.

**Methods:**

Between November 2019 and November 2022, 232 patients operated utilizing supraseptal groove fold-in flap technique using open rhinoplasty approach was retrospectively examined. Supraseptal groove and upper part of the upper lateral cartilages were folded in toward the septum to widen and support the middle vault. Uneven split of the supraseptal groove was performed in favor of the deviation if there was a deviation present. Transcartilaginous horizontal mattress sutures were used if the flap was not stable. Functional results were compared by using Nasal Obstructive Symptoms Evaluation questionnaire whereas subjective Esthetic Appearance test for the esthetic outcomes were administered.

**Results:**

The mean age was 37.1 years (18–63 years), and the mean follow-up time was 18.8 months. Both functional and esthetic outcomes were significantly better in all categories compared to the preoperative assessments (*P* < 0.01).

**Conclusion:**

Supraseptal groove fold-in flap for the reconstruction of middle vault is an effective and promising technique meanwhile preserving as much as cartilage possible in primary rhinoplasties. While widening and supporting mid cartilaginous vault, it can also help to correct the nasal deviation if present.

## Introduction

Rhinoplasty and/or septorhinoplasty operations are becoming more popular while a remarkable percentage of them are solely due to esthetic demands. Therefore, preserving the function of the nose is important while achieving esthetic goals.

Although there are different methods and no gold standard for dorsal reconstruction, back in the day, “T” shaped upper portion of the mid cartilaginous vault is excised if reduction rhinoplasty is intended.^1^ In this technique, early works showed that no significant difference in nasal resistance postoperatively^2^ but latter studies showed significant decrease in cross-sectional area of the nasal vault causing nasal resistance.^3^, ^4^, ^5^ Therefore, the perspective has started to change and the principles of the component dorsal hump reduction technique was adopted.^6^ Incremental reduction of the dorsal septum is performed while preserving upper lateral cartilages; and then different techniques like spreader grafts or upper lateral cartilage fold-in flaps were performed to reconstruct the middle vault while preventing increase in the nasal resistance.^6^, ^7^, ^8^

Preservation rhinoplasty is a popular term; and its importance and implication are becoming more accepted day by day. Preservation instead of resection, replacement of excision by manipulation decrease the need for conchal or costal cartilage grafts for the reconstruction.^9^ As indicated above, this concept was adopted for middle vault reconstruction as well, in other words, the older concept of excision of “T” shaped upper portion is replaced by incremental excision of the solely dorsal septum.^6^ Then the question is raised that can we even spare the dorsal septum and use it to widen the mid cartilaginous vault supporting the internal nasal valve? Can we even use supraseptal groove for rectifying the deviations instead of excising it? In the era of importance sparing even nasal ligaments, should we minimize the septal resection as well?

In this study, we aim to present our novel technique that spare the uppermost part of the septum, supraseptal groove, and use it together with the upper part of the upper lateral cartilages as fold-in flap to widen and support the middle vault. We present our 3-year experience and results with the technique and show its functional and esthetic outcomes.

## Materials and methods

This study was approved by the Institutional Ethical Review Board of a University Medical School (04.11.2023, E-25403353–050.99–2300078413). The study was designed in a retrospective manner and informed consents were obtained from all the patients included in the study. Additional written informed consents for patient information and images to be published were provided by the patients for whom identifying information is included in this article. Medical and personal records of 232 patients operated between November 2019 and November 2022 using supraseptal groove fold-in flap technique were retrospectively assessed. All patients were primary rhinoplasty candidates and operated using open rhinoplasty approach by a single senior surgeon (Y.K.) at a university tertiary care medical center.

Inclusion criteria required at least 3 months of follow-up period, patients being older than 18 years old, pre- and post-operative photographic documentation, and completion of a Nasal Obstructive Symptoms Evaluation (NOSE) questionnaire^10^ which is for the evaluation of functional results and subjective Esthetic Appearance Test^11^ for the esthetic outcomes. Preoperative complaints, surgical indications and desires, intraoperative findings, postoperative complications and complaints like infection, epistaxis, loss of the structural support and tip deprojection and request for a revision surgery were evaluated in detail.

## Surgical technique

232 patients were operated by a single senior surgeon (Y.K.) by using open rhinoplasty approach to provide wide exposure to nasal structures.^12^ All patients underwent surgery under standard general anesthesia. Nasal dorsum, lateral nasal walls, tip, and septum were infiltrated with 1 % lidocaine hydrochloride and 1:100,000 epinephrine for the purpose of vasoconstriction, hydro-dissection and local anesthesia. Transcolumellar and mucosal infracartilaginous incisions were made, and the nasal skin–soft tissue envelope was dissected. Septal exposure was started initially from the anterior septal angle and advanced between upper lateral cartilages and dorsal septum cranially. Bilateral mucoperichondrial flaps were created under the sub-perichondrial plane, and the septum was exposed; meanwhile a great care was taken not to harm mucoperichondrial flaps which could potentially cause cicatricial narrowing of the internal nasal valve.

**Figure 1:**
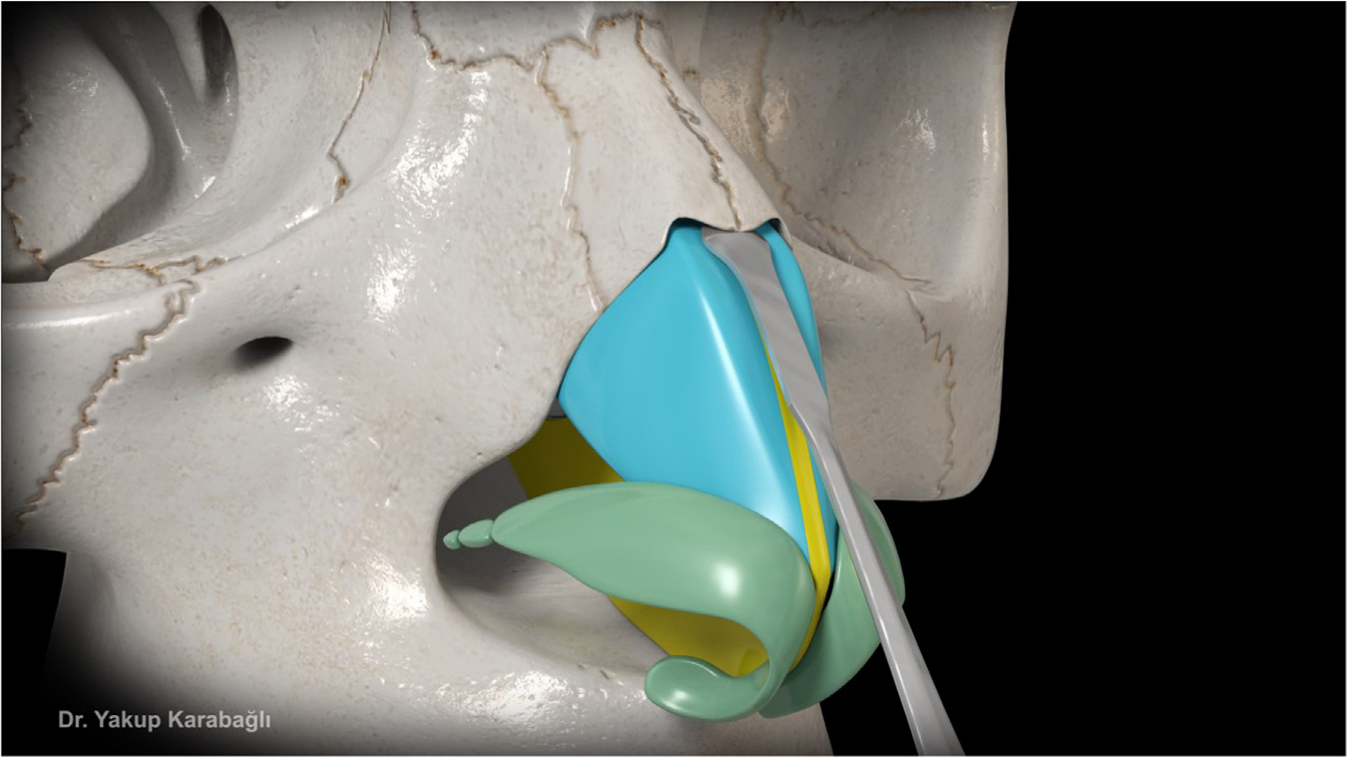
This figure demonstrates dissection of supraseptal groove from the nasal bone at the keystone area with the help of a blunt-tipped dissector.

**Figure 2:**
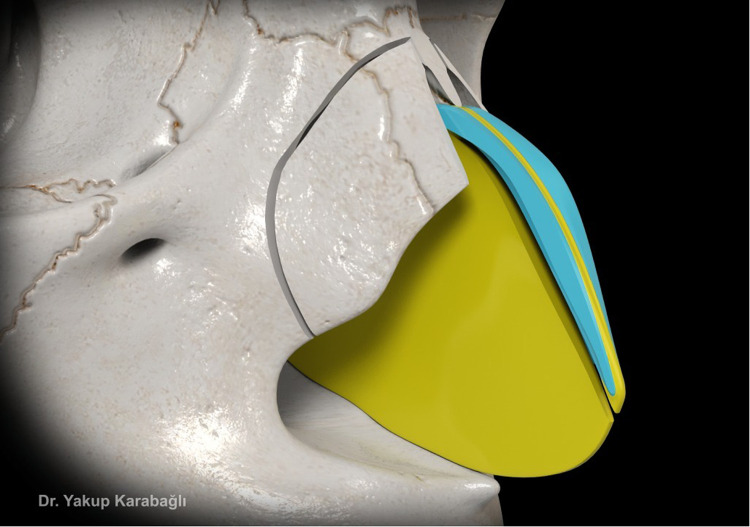
This figure shows the excess dorsal septum that was cut to be incorporated into the folded flaps.

**Figure 3:**
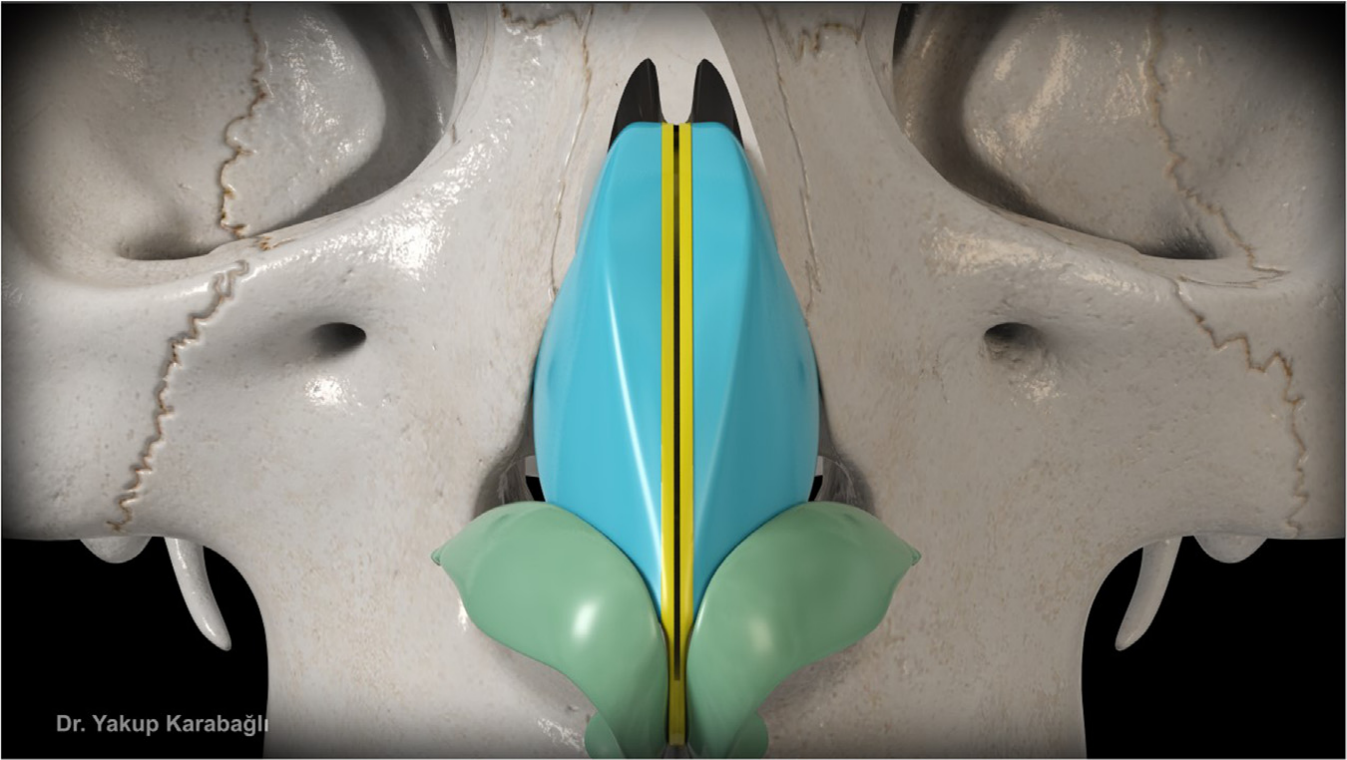
This figure demonstrates midline symmetric incision of supraseptal groove.

**Figure 4:**
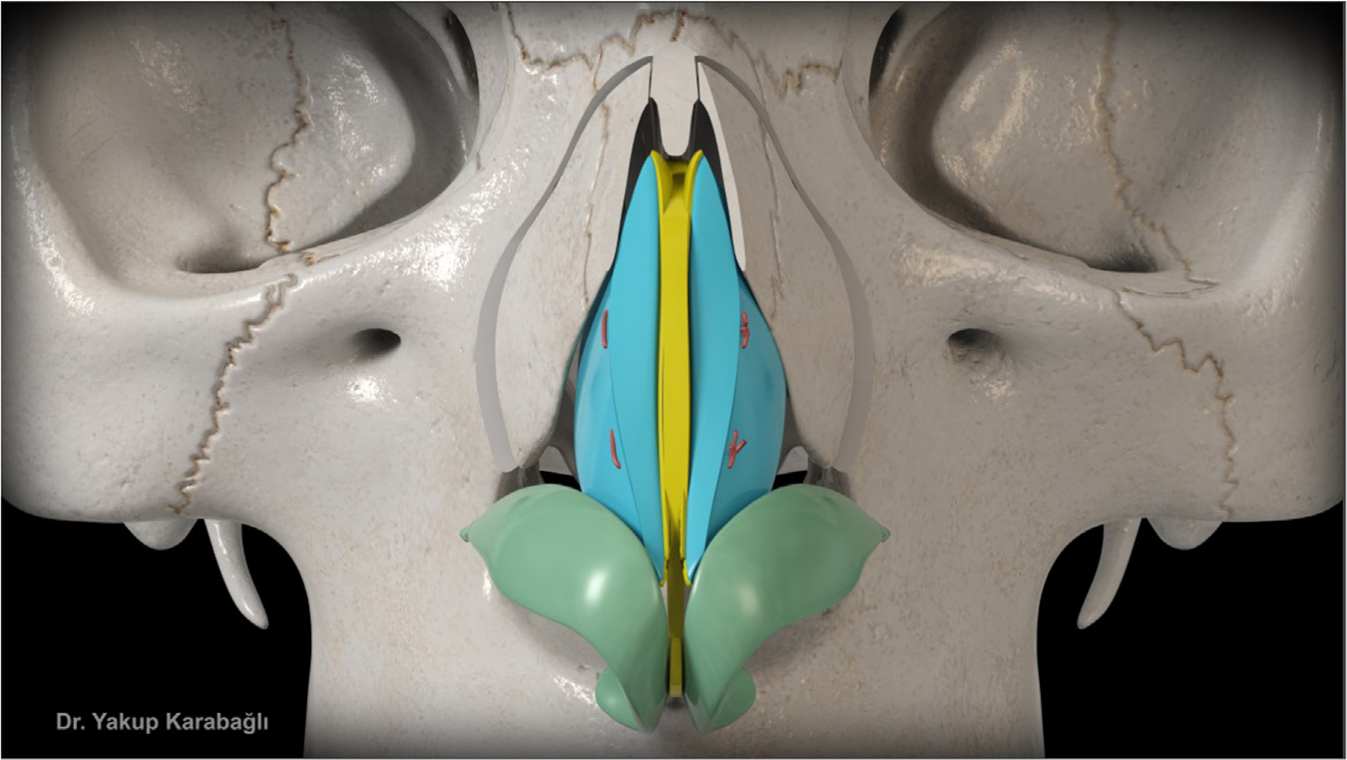
This figure shows transcartilaginous horizontal mattress sutures to hold the flaps in place if the flaps were not stable.

After septal bilateral submucoperichondrial dissection as in component dorsal hump reduction technique,^6^ upper lateral cartilages and supraseptal groove were separated from the nasal bone at the keystone area with the help of a blunt-tipped dissector (Figure 1). Dorsal bony reduction was carried out with double-guarded osteotome and nasal rasps or piezo-electrical devices. Routine medial and lateral osteotomies were performed if required. Afterwards, instead of incremental excision of supraseptal groove and transverse portion of upper lateral cartilages as in component dorsal hump reduction technique,^6^ they were preserved to be used as a fold-in flap for the reconstruction of middle vault. For this purpose, excess dorsal septum was trimmed with angled septum scissors but not excised (Figure 2). Then, supraseptal groove was split into two creating two flaps consisting of supraseptal groove and transverse portion of upper lateral cartilages. These are called flaps but not grafts since they were still attached to the upper lateral cartilages but also the flaps could easily be molded. If there was a deviation of the nose present, supraseptal groove was incised away from the deviated side (Video-1) but if no deviation is present, it was incised in the middle (Figure 3). Afterwards, these created flaps were folded inwards at both sides of the nasal septum (Video-2). As indicated before, if there was a deviation present, the larger flap was used in favor of the deviation to push the septum to midline. Transcartilaginous horizontal mattress sutures were used if the flaps were not stable (Figure 4) but the suturation was not necessary if the flaps were not sliding from where they were placed.

After management of cartilaginous hump meanwhile supporting mid cartilaginous vault with the fold-in flaps, tip-plasty was performed, and all incisions were closed properly. A plaster cast and intranasal Doyle splint was applied, and the procedure was terminated (Supplementary Video-1).

## Statistical analysis

The data were analyzed using IBM SPSS 21.0 (IBM Corp., Armonk, NY, USA). Shapiro Wilk’s test was used to determine the suitability of the variables for normal distribution. Parametric and non-parametric tests were used to compare the groups. Independent samples t-test and Mann Whitney U test were used to compare independent groups according to distribution forms. Chi-square tests were used in the analysis of cross-tabulations. Marginal homogeneity test was used to compare preoperative and postoperative values. For the presentation of the data, number (%) was used for qualitative data and Mean ± SD or Median (Q1; Q3) values ​​were used for quantitative data. The significance level was set at *P* < 0.05.

## Results

The youngest patient was 18 years old while the eldest was 63 years of age, the mean age being 37.1 ± 13.2 years. The mean follow-up time was 18.8 ± 7.7 months ranging from 5 months to 3 years (Supplementary Table 1). All patients had their primary rhinoplasty procedures, secondary rhinoplasty cases were not included. Most of the patients had breathing problems of varying degrees and all of them had different esthetic concerns about their noses.

Pre- and post-operative functional results were evaluated by NOSE questionnaire^10^ (Table 1) and statistical analyses were performed to compare the results. Statistically significant improvements were observed in all functional categories (Supplementary Table 2).

**Table 1:** Nasal obstructive symptoms evaluation (NOSE) questionnaire.^10^

	Not a problem	Very mild	Moderate	Fairly bad	Severe
1. Nasal congestion and stuffiness	0	1	2	3	4
2. Nasal blockage and obstruction	0	1	2	3	4
3. Trouble breathing through my nose	0	1	2	3	4
4. Trouble sleeping	0	1	2	3	4
5. Unable to get enough air through my nose during exercise and exertion	0	1	2	3	4

**Figure 5:**
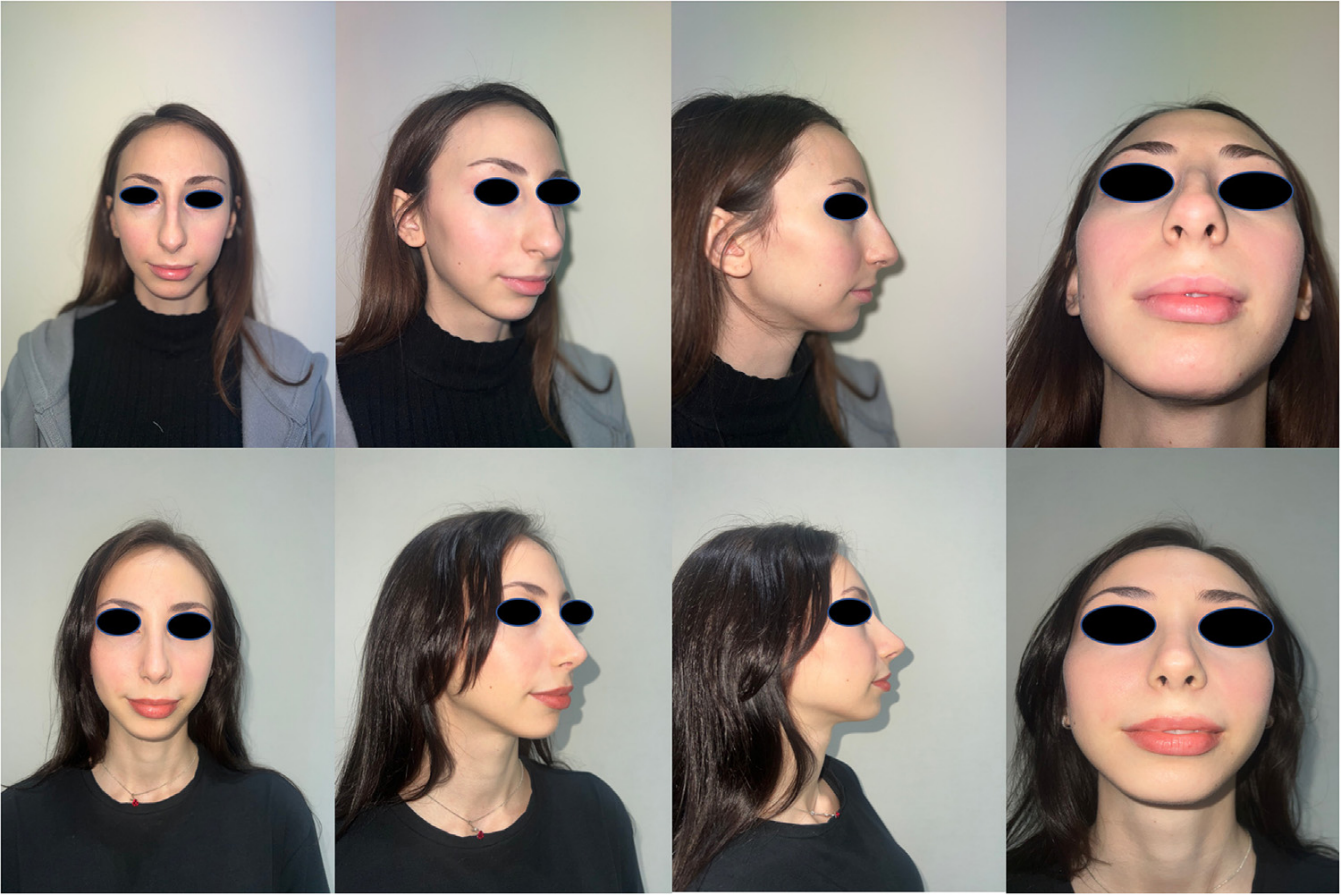
Pre- and post-operative results of a 19-year-old female patient.

**Figure 6:**
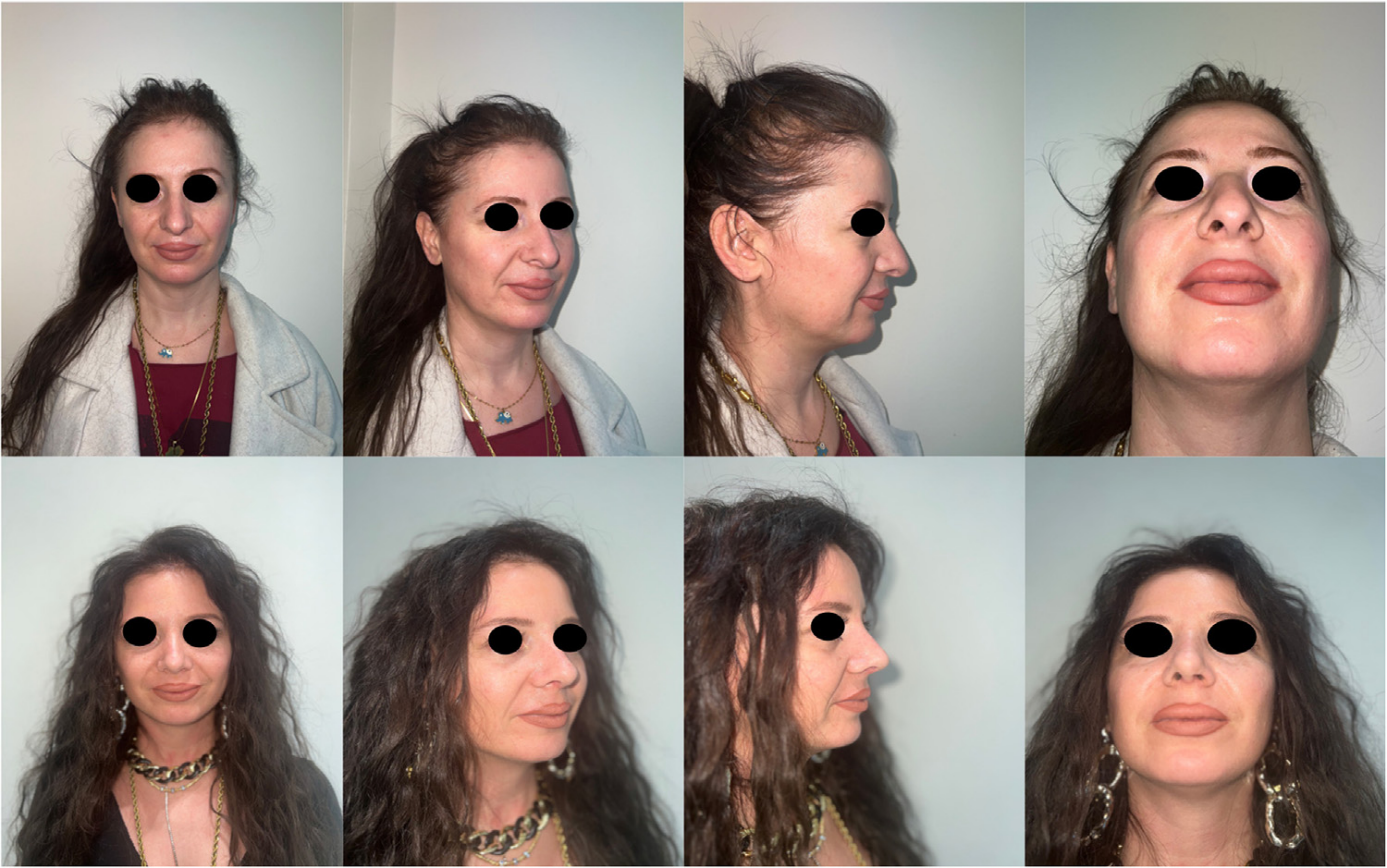
Pre- and post-operative results of a 52-year-old female patient.

On the other hand, significant satisfaction was observed about the esthetic outcomes, as well, as shown in Table 2 and Supplementary Table 3. None of the patients complained about inverted-V deformity or showed the signs of middle-vault narrowing. Only 12 patients requested a revision surgery due to tip deprojection that occurred during the follow-ups. However, none of the patients requested a revision surgery or complained about any dorsal irregularities. Overall, a stable appearance of the dorsum and the nose indicated the success of the surgical technique (Figure 5, Figure 6).

**Table 2:** Subjective esthetic appearance test.^11^

	None	Mild	Moderate	Severe
1. Deprojection	0	1	2	3
2. Pseudo-hump appearance	0	1	2	3
3. Tip definition	0	1	2	3
4- Unsatisfaction	0	1	2	3

No major complications or complaints were seen but only common complaints were expressed as in routine rhinoplasty procedures such as facial swelling, nasal stuffiness, pain, and/or epistaxis. Almost all of these complaints were resolved postoperatively by head elevation and cold application.

## Discussion

Demand for the esthetic procedures are on the rise and the rhinoplasty procedures are one of the most popular ones in the list.^13^ Not only providing better esthetic outcomes is important but also preserving the functionality and securing the nasal airway with normal breathing is crucial. One of the key goals for this purpose is to reconstruct the middle vault in order not to increase the nasal resistance.

There are different methods for the reconstruction of the nasal dorsum during primary rhinoplasty procedures. Although early practices were mainly relied on excisions intending reduction rhinoplasties,^1^ in time, preservation of the structures become popular and has been adopted by the most.^9^ Since the nasal resistance is increased and caused trouble breathing in early surgical practices,^3^, ^4^, ^5^ principles of the component dorsal hump reduction technique were acknowledged and incremental reduction while preserving upper lateral cartilages has been performed.^6^ Afterwards, reconstruction with spreader grafts^8^ or upper lateral cartilage fold-in-flaps techniques^7^ were carried out in order to restore the middle vault and not to increase nasal resistance. However, to the best of our knowledge, there is no literature present yet using supraseptal groove flap for the reconstruction of the middle vault. In the present study, supraseptal groove and transverse portion of upper lateral cartilages were used in a fold-in-flap fashion to reconstruct and widen the middle vault while even rectifying the dorsal deviations when the flap was designed asymmetrically.

Two details deserve special attention in this surgical technique. First, the deviation should be detected initially and if present, the incision should be planned in favor of the deviation in order to push the septum to the midline with the fold-in-flap. Second, the flaps should be detached even under the keystone area to be able to fold in easily but not from the upper lateral cartilages in order not to detach it completely causing formation of a graft rather than a flap.

Functional results of 232 patients were evaluated in this study showing improvement in nasal breathing according to the NOSE questionnaire. In all categories of the NOSE questionnaire, lower scores were obtained postoperatively. Although the questions and the answers to them are still subjective, the NOSE questionnaire has been used many times by other authors suggesting its wide acceptance and legitimacy.^14^, ^15^, ^16^, ^17^ As a result, our novel surgical technique presents promising results in fixing breathing problems and better functional outcomes.

For the esthetic outcomes, subjective Esthetic Appearance Test was used.^11^ Deprojection, pseudo-hump appearance, tip definition and overall unsatisfaction are the items of this test. In all categories of the test, statistically significant lower scores, therefore, more satisfied outcomes were obtained postoperatively. Especially for the pseudo-hump appearance question, only 4 patients gave higher values compared to preoperative assessment indicating a stable and smooth dorsum achieved with this technique. Furthermore, only 12 patients had higher unsatisfaction scores compared to preoperative assessments scores which is another indicator of the success of the technique.

No major complications were seen after the surgeries in any of the patients including infection, hematoma, skin necrosis or graft extrusion, resorption or dislocation. The main complaints were consistent with the complaints seen in classic rhinoplasty procedures including epistaxis, facial swelling, pain and nasal stuffiness. These complaints were resolved without any intervention within two weeks. Only 12 patients requested revision surgery for tip deprojection diagnosed during follow-ups, no other revision surgery is planned or performed for any other complaints. Overall, these results show satisfying functional and esthetic outcomes with a safe and reliable technique.

The limitations of this study include its application for primary cases, retrospective design and subjective assessments of the functional and esthetic results. Although this technique can be widely applicable for primary rhinoplasty cases, in secondary cases, it may not be possible to implement the technique due to possible previous excision of supraseptal groove and transverse part of the upper lateral cartilages. Furthermore, even if NOSE questionnaire is widely used and accepted by other authors,^14^, ^15^, ^16^, ^17^ as well, it is still a subjective test based on answers of the patients compared to more objective tests like acoustic rhinometry^18^ or rhinomanometry.^19^ For the esthetic outcomes, there is no objective tests or scoring systems in the literature to the best of our knowledge. Another limitation might be considered as not including a control or comparison group using a different surgical technique. Therefore, conducting a prospective study with a control group using a different surgical technique might give us better outcomes with more precise results.

## Conclusion

We present a novel technique preserving and using supraseptal groove and transverse portion of upper lateral cartilages as a flap for widening and supporting the middle vault. Furthermore, if a deviation is present, uneven split of the flap might assist overcoming the deviations. The technique is reliable and safe, and can be used in primary rhinoplasty procedures successfully.

## Ethical approval

All procedures followed were in accordance with the ethical standards of the responsible committee on human experimentation (institutional and national) and with the Helsinki Declaration of 1975, as revised in 2013. Furthermore, this study was approved by the Institutional Ethical Review Board of a University Medical School (04.11.2023, E-25403353–050.99–2300078413). Informed consents were obtained from all the patients included in the study. Additional written informed consents for patient information and images to be published were provided by the patients for whom identifying information is included in this article.

## Funding

None.

## Declaration of competing interest

None declared.

## Appendix Supplementary materials

Supplementary Table 1: General patient features Image, application 1

Supplementary Table 2: Results of Nasal Obstructive Symptoms Evaluation (NOSE) questionnaire Image, application 2

Supplementary Table 3: Results of subjective esthetic appearance Image, application 3

Video 1: This video shows asymmetric incision of supraseptal groove away from the deviated side. Image, video 4

Video 2: This video demonstrates folding the flaps inwards at both sides of the nasal septum to widen the middle vault Image, video 5

## References

[1] Pollet J., Weikel A. (1976). Some technical considerations in primary rhinoplasty. Aesthetic Plast Surg.

[2] Adamson P., Smith O., Cole P. (1990). The effect of cosmetic rhinoplasty on nasal patency. Laryngoscope.

[3] Grymer L.F (1995). Reduction rhinoplasty and nasal patency: change in the cross-sectional area of the nose evaluated by acoustic rhinometry. Laryngoscope.

[4] Beekhuis G.J (1976). Nasal obstruction after rhinoplasty: etiology, and techniques for correction. Laryngoscope.

[5] Goldman I.B (1966). Rhinoplastic sequelae causing nasal obstruction. Arch Otolaryngol.

[6] Rohrich R.J., Muzaffar A.R., Janis J.E (2004). Component dorsal hump reduction: the importance of maintaining dorsal aesthetic lines in rhinoplasty. Plast Reconstr Surg.

[7] Ozmen S., Ayhan S., Findikcioglu K., Kandal S., Atabay K. (2008). Upper lateral cartilage fold-in flap: a combined spreader and/or splay graft effect without cartilage grafts. Ann Plast Surg.

[8] Sheen J.H (1984). Spreader graft: a method of reconstructing the roof of the middle nasal vault following rhinoplasty. Plast Reconstr Surg.

[9] Daniel R.K (2018). The preservation rhinoplasty: a new rhinoplasty revolution. Aesthet Surg J.

[10] Stewart M.G., Smith T.L., Weaver E.M. (2004). Outcomes after nasal septoplasty: results from the Nasal Obstruction Septoplasty Effectiveness (NOSE) study. Otolaryngol Head Neck Surg.

[11] Ekinci C., Can B., Koçman A. (2022). Dorsocaudal reconstruction of previous caudal septal resections with partial split spreader graft. J Surg Med.

[12] Rohrich R.J., Gunter J.P., Deuber M.A., Adams W.P. (2002). The deviated nose: optimizing results using a simplified classification and algorithmic approach. Plast Reconstr Surg.

[13] Fichman M., Piedra Buena I.T. (2023). StatPearls.

[14] Karahatay S., Taşlı H., Karakoç Ö., Aydın Ü., Türker T. (2018). Reliability and validity of the Turkish Nose obstruction symptom evaluation (NOSE) scale. Turk J Med Sci.

[15] Spiekermann C., Savvas E., Rudack C., Stenner M. (2018). Adaption and validation of the nasal obstruction symptom evaluation scale in German language (D-NOSE). Health Qual Life Outcomes.

[16] Balsevicius T., Padervinskis E., Pribuisiene R., Kuzminiene A., Vaitkus S., Liutkevicius V. (2021). Cross-cultural adaptation and validation of Lithuanian-NOSE scale. Eur Arch Otorhinolaryngol.

[17] Marro M., Mondina M., Stoll D., de Gabory L. (2011). French validation of the NOSE and RhinoQOL questionnaires in the management of nasal obstruction. Otolaryngol: Head Neck Surg.

[18] Roithmann R., Cole P., Chapnik J., Shpirer I., Hoffstein V., Zamel N. (1995). Acoustic rhinometry in the evaluation of nasal obstruction. Laryngoscope.

[19] Demirbas D., Cingi C., Çakli H., Kaya E. (2011). Use of rhinomanometry in common rhinologic disorders. Expert Rev Med Devices.

