# Supplementary material for: Supraseptal groove fold-in flap for the reconstruction of middle vault in primary rhinoplasty
Source: JPRAS Open. 2025 Jun 18;45:251–9. doi: 10.1016/j.jpra.2025.06.005 (PMC12275147; doi:10.1016/j.jpra.2025.06.005)
Supplement: Supplementary file 1 — Supplementary Table 1: General patient features [file mmc1.docx]

Supplementary Table 1.: General patient features

| Patient No. | Age | Gender | Follow-Up Duration (Month) | Split Type | Revision Surgery |
| --- | --- | --- | --- | --- | --- |
| 1 | 22 | F | 36 | S | N |
| 2 | 37 | F | 36 | S | N |
| 3 | 44 | M | 35 | S | N |
| 4 | 19 | F | 35 | S | N |
| 5 | 57 | M | 35 | AS | N |
| 6 | 43 | F | 35 | S | N |
| 7 | 18 | F | 34 | S | N |
| 8 | 51 | F | 34 | AS | N |
| 9 | 33 | M | 34 | S | N |
| 10 | 47 | M | 34 | S | N |
| 11 | 27 | F | 33 | S | N |
| 12 | 20 | M | 33 | S | N |
| 13 | 43 | F | 32 | S | N |
| 14 | 37 | F | 32 | S | N |
| 15 | 22 | M | 32 | AS | N |
| 16 | 54 | F | 32 | AS | N |
| 17 | 19 | M | 32 | S | N |
| 18 | 33 | F | 31 | S | N |
| 19 | 37 | M | 31 | S | Y |
| 20 | 44 | M | 31 | S | N |
| 21 | 39 | F | 30 | AS | N |
| 22 | 29 | F | 30 | AS | N |
| 23 | 25 | F | 30 | AS | N |
| 24 | 48 | M | 30 | S | N |
| 25 | 61 | F | 30 | S | N |
| 26 | 47 | M | 30 | AS | N |
| 27 | 38 | M | 29 | AS | N |
| 28 | 32 | M | 29 | AS | N |
| 29 | 48 | F | 29 | S | N |
| 30 | 26 | M | 28 | S | N |
| 31 | 31 | F | 28 | S | N |
| 32 | 57 | F | 28 | S | N |
| 33 | 63 | M | 27 | AS | N |
| 34 | 55 | F | 27 | AS | N |
| 35 | 43 | M | 27 | AS | N |
| 36 | 52 | F | 27 | S | N |
| 37 | 19 | M | 27 | AS | N |
| 38 | 38 | F | 27 | AS | N |
| 39 | 28 | F | 26 | AS | N |
| 40 | 56 | M | 26 | AS | N |
| 41 | 60 | F | 26 | AS | N |
| 42 | 27 | M | 26 | S | Y |
| 43 | 30 | F | 26 | S | N |
| 44 | 44 | F | 26 | S | N |
| 45 | 18 | M | 26 | AS | N |
| 46 | 31 | M | 26 | AS | N |
| 47 | 55 | F | 26 | AS | N |
| 48 | 62 | M | 25 | S | N |
| 49 | 22 | F | 25 | AS | N |
| 50 | 29 | F | 25 | AS | N |
| 51 | 40 | F | 25 | S | Y |
| 52 | 43 | M | 25 | AS | N |
| 53 | 20 | F | 25 | S | N |
| 54 | 27 | M | 25 | AS | N |
| 55 | 56 | M | 25 | S | N |
| 56 | 31 | M | 25 | S | N |
| 57 | 24 | F | 25 | AS | N |
| 58 | 36 | F | 25 | AS | N |
| 59 | 55 | M | 24 | S | N |
| 60 | 25 | F | 24 | AS | N |
| 61 | 21 | F | 24 | S | N |
| 62 | 20 | F | 24 | AS | N |
| 63 | 55 | F | 24 | S | N |
| 64 | 53 | F | 24 | AS | N |
| 65 | 39 | M | 24 | AS | Y |
| 66 | 22 | F | 24 | AS | N |
| 67 | 30 | M | 24 | S | N |
| 68 | 27 | F | 24 | S | N |
| 69 | 49 | M | 23 | S | N |
| 70 | 41 | F | 23 | AS | N |
| 71 | 60 | F | 23 | S | N |
| 72 | 32 | M | 23 | AS | N |
| 73 | 56 | F | 22 | S | N |
| 74 | 22 | F | 22 | AS | N |
| 75 | 42 | M | 22 | S | N |
| 76 | 28 | F | 22 | AS | N |
| 77 | 57 | F | 22 | AS | N |
| 78 | 48 | M | 22 | AS | N |
| 79 | 20 | M | 22 | AS | N |
| 80 | 39 | F | 22 | S | N |
| 81 | 40 | F | 22 | AS | N |
| 82 | 62 | F | 22 | S | N |
| 83 | 38 | M | 22 | AS | Y |
| 84 | 29 | F | 21 | AS | N |
| 85 | 58 | M | 21 | S | N |
| 86 | 33 | F | 21 | S | N |
| 87 | 49 | M | 21 | AS | N |
| 88 | 53 | F | 21 | S | N |
| 89 | 41 | F | 21 | S | N |
| 90 | 26 | F | 21 | S | N |
| 91 | 20 | M | 21 | AS | N |
| 92 | 32 | F | 21 | AS | N |
| 93 | 59 | M | 21 | S | N |
| 94 | 18 | M | 20 | AS | N |
| 95 | 25 | F | 20 | S | N |
| 96 | 44 | M | 20 | AS | N |
| 97 | 23 | F | 20 | AS | N |
| 98 | 39 | F | 20 | S | N |
| 99 | 51 | F | 20 | AS | N |
| 100 | 47 | M | 20 | AS | N |
| 101 | 40 | F | 20 | AS | Y |
| 102 | 29 | F | 20 | AS | N |
| 103 | 33 | M | 19 | S | N |
| 104 | 21 | F | 19 | S | N |
| 105 | 27 | M | 19 | AS | N |
| 106 | 36 | M | 19 | S | N |
| 107 | 59 | F | 19 | AS | N |
| 108 | 22 | M | 19 | S | N |
| 109 | 40 | F | 19 | AS | N |
| 110 | 53 | M | 19 | S | N |
| 111 | 24 | F | 19 | AS | N |
| 112 | 50 | M | 18 | AS | N |
| 113 | 27 | M | 18 | S | N |
| 114 | 41 | F | 18 | S | N |
| 115 | 39 | F | 18 | AS | N |
| 116 | 18 | M | 18 | S | N |
| 117 | 58 | F | 18 | S | N |
| 118 | 44 | F | 18 | AS | N |
| 119 | 63 | F | 18 | AS | N |
| 120 | 22 | M | 18 | S | N |
| 121 | 31 | F | 18 | S | N |
| 122 | 25 | F | 18 | AS | N |
| 123 | 43 | M | 18 | S | N |
| 124 | 55 | M | 18 | S | N |
| 125 | 37 | F | 18 | S | N |
| 126 | 20 | F | 17 | AS | Y |
| 127 | 47 | F | 17 | AS | N |
| 128 | 26 | M | 17 | AS | N |
| 129 | 35 | F | 17 | S | N |
| 130 | 60 | M | 17 | S | N |
| 131 | 51 | F | 17 | AS | N |
| 132 | 23 | M | 17 | AS | N |
| 133 | 56 | M | 17 | S | N |
| 134 | 20 | F | 17 | AS | N |
| 135 | 33 | F | 17 | S | N |
| 136 | 19 | M | 17 | AS | N |
| 137 | 58 | M | 17 | S | N |
| 138 | 36 | F | 17 | AS | N |
| 139 | 25 | F | 16 | AS | N |
| 140 | 38 | F | 16 | S | N |
| 141 | 52 | F | 16 | AS | N |
| 142 | 30 | M | 16 | AS | N |
| 143 | 59 | F | 16 | S | N |
| 144 | 61 | M | 16 | AS | N |
| 145 | 23 | F | 15 | AS | N |
| 146 | 42 | F | 15 | S | N |
| 147 | 50 | M | 15 | AS | N |
| 148 | 50 | F | 15 | AS | N |
| 149 | 27 | F | 15 | S | N |
| 150 | 48 | M | 15 | S | Y |
| 151 | 33 | F | 15 | AS | N |
| 152 | 29 | M | 15 | S | N |
| 153 | 40 | F | 15 | AS | N |
| 154 | 53 | F | 15 | AS | N |
| 155 | 61 | M | 15 | S | N |
| 156 | 29 | F | 15 | S | N |
| 157 | 20 | F | 15 | AS | N |
| 158 | 31 | F | 14 | AS | N |
| 159 | 22 | M | 14 | AS | N |
| 160 | 49 | M | 14 | S | N |
| 161 | 40 | F | 14 | S | N |
| 162 | 20 | M | 14 | AS | N |
| 163 | 33 | F | 14 | S | N |
| 164 | 47 | F | 14 | S | N |
| 165 | 21 | M | 14 | AS | N |
| 166 | 51 | F | 14 | S | Y |
| 167 | 38 | F | 14 | S | N |
| 168 | 19 | M | 14 | AS | N |
| 169 | 27 | M | 14 | AS | N |
| 170 | 44 | F | 14 | S | N |
| 171 | 31 | M | 14 | AS | N |
| 172 | 18 | F | 13 | S | N |
| 173 | 29 | M | 13 | AS | N |
| 174 | 46 | F | 13 | AS | N |
| 175 | 22 | F | 13 | AS | N |
| 176 | 38 | F | 13 | S | N |
| 177 | 61 | M | 13 | AS | N |
| 178 | 40 | F | 13 | S | N |
| 179 | 38 | F | 12 | AS | N |
| 180 | 26 | M | 12 | AS | N |
| 181 | 21 | F | 12 | S | N |
| 182 | 27 | F | 12 | S | N |
| 183 | 45 | F | 12 | AS | N |
| 184 | 30 | M | 12 | AS | Y |
| 185 | 55 | M | 12 | S | N |
| 186 | 21 | F | 12 | S | N |
| 187 | 48 | M | 12 | AS | N |
| 188 | 28 | F | 12 | S | N |
| 189 | 20 | F | 11 | AS | N |
| 190 | 32 | M | 11 | AS | N |
| 191 | 43 | F | 11 | AS | N |
| 192 | 52 | M | 11 | S | N |
| 193 | 19 | F | 11 | S | N |
| 194 | 28 | F | 10 | S | N |
| 195 | 41 | F | 10 | AS | N |
| 196 | 39 | M | 10 | S | N |
| 197 | 20 | M | 10 | AS | Y |
| 198 | 57 | F | 10 | AS | N |
| 199 | 36 | M | 10 | S | N |
| 200 | 24 | F | 10 | AS | N |
| 201 | 46 | M | 10 | S | N |
| 202 | 18 | F | 9 | AS | N |
| 203 | 51 | M | 9 | S | N |
| 204 | 33 | F | 9 | AS | N |
| 205 | 23 | M | 9 | S | N |
| 206 | 46 | M | 9 | S | N |
| 207 | 59 | F | 9 | S | N |
| 208 | 30 | M | 9 | AS | N |
| 209 | 42 | F | 9 | AS | N |
| 210 | 23 | M | 8 | AS | N |
| 211 | 51 | F | 8 | AS | N |
| 212 | 62 | F | 8 | S | Y |
| 213 | 22 | F | 8 | AS | N |
| 214 | 28 | M | 8 | S | N |
| 215 | 39 | M | 8 | AS | N |
| 216 | 20 | F | 8 | AS | N |
| 217 | 50 | M | 7 | S | N |
| 218 | 26 | F | 7 | AS | N |
| 219 | 21 | M | 7 | S | N |
| 220 | 43 | M | 7 | AS | N |
| 221 | 34 | F | 7 | S | N |
| 222 | 46 | F | 6 | AS | N |
| 223 | 18 | F | 6 | S | N |
| 224 | 37 | M | 6 | AS | N |
| 225 | 22 | F | 6 | S | N |
| 226 | 51 | M | 6 | AS | N |
| 227 | 43 | F | 6 | AS | N |
| 228 | 20 | M | 5 | S | N |
| 229 | 31 | F | 5 | AS | N |
| 230 | 46 | M | 5 | S | N |
| 231 | 19 | F | 5 | S | N |
| 232 | 22 | F | 5 | AS | N |

F: Female, M: Male, S: Symmetrical, AS: Asymmetrical, N: No, Y: Yes
