# Supplementary material for: Supraseptal groove fold-in flap for the reconstruction of middle vault in primary rhinoplasty
Source: JPRAS Open. 2025 Jun 18;45:251–9. doi: 10.1016/j.jpra.2025.06.005 (PMC12275147; doi:10.1016/j.jpra.2025.06.005)
Supplement: Supplementary file 3 — Supplementary Table 3: Results of subjective esthetic appearance [file mmc3.docx]

Supplementary Table 3.: Results of Subjective Esthetic Appearance

|  | Deprojection | | Pseudo-hump  Appearance | | Tip Definition | | | | Unsatisfaction | |
| --- | --- | --- | --- | --- | --- | --- | --- | --- | --- | --- |
| Patient | Pre | Post | Pre | Post | Pre | | Post | | Pre | Post |
| 1 | 2 | 0 | 2 | 0 | 3 | 1 | | 3 | | 0 |
| 2 | 3 | 1 | 2 | 0 | 3 | 1 | | 3 | | 1 |
| 3 | 1 | 0 | 2 | 0 | 1 | 0 | | 2 | | 0 |
| 4 | 3 | 0 | 2 | 1 | 3 | 1 | | 3 | | 1 |
| 5 | 2 | 0 | 1 | 0 | 3 | 1 | | 2 | | 0 |
| 6 | 2 | 0 | 2 | 1 | 2 | 1 | | 2 | | 0 |
| 7 | 3 | 1 | 2 | 1 | 3 | 2 | | 2 | | 1 |
| 8 | 2 | 0 | 3 | 0 | 2 | 0 | | 3 | | 0 |
| 9 | 3 | 0 | 2 | 1 | 3 | 0 | | 3 | | 1 |
| 10 | 2 | 2 | 1 | 2 | 2 | 2 | | 1 | | 2 |
| 11 | 3 | 0 | 3 | 0 | 3 | 0 | | 3 | | 0 |
| 12 | 2 | 1 | 1 | 1 | 2 | 1 | | 2 | | 1 |
| 13 | 3 | 1 | 2 | 1 | 2 | 0 | | 2 | | 0 |
| 14 | 2 | 0 | 1 | 0 | 2 | 0 | | 2 | | 0 |
| 15 | 3 | 1 | 2 | 0 | 3 | 0 | | 3 | | 0 |
| 16 | 3 | 0 | 3 | 0 | 3 | 0 | | 3 | | 0 |
| 17 | 2 | 1 | 2 | 0 | 2 | 0 | | 2 | | 0 |
| 18 | 1 | 1 | 2 | 1 | 2 | 1 | | 2 | | 1 |
| 19 | 2 | 3 | 1 | 3 | 1 | 3 | | 1 | | 3 |
| 20 | 2 | 0 | 3 | 0 | 2 | 0 | | 3 | | 0 |
| 21 | 3 | 1 | 3 | 0 | 3 | 0 | | 3 | | 1 |
| 22 | 3 | 1 | 3 | 0 | 2 | 0 | | 3 | | 0 |
| 23 | 2 | 0 | 2 | 0 | 3 | 0 | | 2 | | 0 |
| 24 | 2 | 1 | 1 | 0 | 1 | 0 | | 2 | | 0 |
| 25 | 1 | 1 | 1 | 0 | 1 | 1 | | 1 | | 1 |
| 26 | 2 | 0 | 1 | 0 | 2 | 0 | | 2 | | 0 |
| 27 | 2 | 1 | 2 | 0 | 2 | 0 | | 2 | | 0 |
| 28 | 3 | 0 | 3 | 1 | 3 | 1 | | 3 | | 1 |
| 29 | 3 | 0 | 3 | 0 | 2 | 0 | | 3 | | 0 |
| 30 | 2 | 2 | 2 | 1 | 2 | 2 | | 2 | | 2 |
| 31 | 3 | 1 | 3 | 0 | 3 | 1 | | 3 | | 1 |
| 32 | 2 | 0 | 3 | 0 | 3 | 1 | | 2 | | 0 |
| 33 | 3 | 0 | 3 | 0 | 3 | 1 | | 3 | | 0 |
| 34 | 2 | 0 | 2 | 0 | 3 | 1 | | 2 | | 0 |
| 35 | 3 | 0 | 3 | 0 | 3 | 0 | | 3 | | 0 |
| 36 | 2 | 1 | 2 | 0 | 2 | 1 | | 2 | | 1 |
| 37 | 3 | 1 | 3 | 0 | 3 | 1 | | 3 | | 1 |
| 38 | 2 | 2 | 2 | 1 | 2 | 1 | | 2 | | 2 |
| 39 | 1 | 0 | 2 | 0 | 1 | 0 | | 2 | | 0 |
| 40 | 2 | 1 | 1 | 1 | 2 | 2 | | 2 | | 1 |
| 41 | 2 | 2 | 2 | 1 | 2 | 2 | | 2 | | 2 |
| 42 | 1 | 3 | 2 | 1 | 1 | 3 | | 1 | | 2 |
| 43 | 2 | 1 | 2 | 0 | 2 | 1 | | 2 | | 1 |
| 44 | 2 | 1 | 2 | 1 | 2 | 0 | | 2 | | 1 |
| 45 | 2 | 0 | 3 | 1 | 3 | 0 | | 3 | | 0 |
| 46 | 3 | 1 | 3 | 0 | 3 | 1 | | 3 | | 1 |
| 47 | 1 | 1 | 2 | 1 | 1 | 1 | | 1 | | 1 |
| 48 | 2 | 0 | 3 | 0 | 2 | 1 | | 2 | | 0 |
| 49 | 3 | 0 | 3 | 0 | 3 | 0 | | 3 | | 0 |
| 50 | 2 | 1 | 2 | 0 | 2 | 1 | | 2 | | 1 |
| 51 | 2 | 3 | 1 | 1 | 1 | 3 | | 2 | | 3 |
| 52 | 3 | 1 | 3 | 0 | 3 | 1 | | 3 | | 1 |
| 53 | 2 | 1 | 1 | 1 | 2 | 2 | | 2 | | 2 |
| 54 | 2 | 2 | 2 | 1 | 2 | 2 | | 2 | | 1 |
| 55 | 2 | 0 | 3 | 0 | 2 | 0 | | 2 | | 0 |
| 56 | 3 | 0 | 3 | 1 | 3 | 0 | | 3 | | 0 |
| 57 | 2 | 0 | 2 | 0 | 2 | 0 | | 2 | | 0 |
| 58 | 3 | 0 | 2 | 0 | 3 | 1 | | 2 | | 0 |
| 59 | 2 | 1 | 2 | 0 | 2 | 1 | | 2 | | 1 |
| 60 | 3 | 0 | 3 | 0 | 2 | 0 | | 3 | | 0 |
| 61 | 1 | 2 | 1 | 0 | 1 | 2 | | 1 | | 1 |
| 62 | 3 | 0 | 3 | 0 | 3 | 0 | | 3 | | 0 |
| 63 | 2 | 1 | 3 | 1 | 2 | 1 | | 2 | | 1 |
| 64 | 3 | 1 | 3 | 0 | 3 | 1 | | 3 | | 1 |
| 65 | 2 | 2 | 2 | 1 | 2 | 3 | | 2 | | 2 |
| 66 | 2 | 1 | 2 | 1 | 2 | 0 | | 2 | | 1 |
| 67 | 2 | 0 | 3 | 0 | 2 | 1 | | 2 | | 0 |
| 68 | 2 | 2 | 3 | 1 | 2 | 1 | | 2 | | 1 |
| 69 | 3 | 0 | 3 | 1 | 3 | 1 | | 3 | | 1 |
| 70 | 2 | 0 | 2 | 0 | 3 | 1 | | 2 | | 0 |
| 71 | 2 | 1 | 2 | 0 | 2 | 1 | | 2 | | 1 |
| 72 | 2 | 0 | 3 | 1 | 2 | 0 | | 2 | | 0 |
| 73 | 2 | 1 | 2 | 0 | 2 | 0 | | 2 | | 1 |
| 74 | 3 | 0 | 3 | 0 | 3 | 1 | | 3 | | 1 |
| 75 | 2 | 0 | 3 | 0 | 2 | 0 | | 3 | | 0 |
| 76 | 2 | 2 | 2 | 1 | 2 | 2 | | 2 | | 2 |
| 77 | 3 | 1 | 3 | 0 | 3 | 0 | | 3 | | 0 |
| 78 | 2 | 1 | 2 | 0 | 2 | 1 | | 2 | | 1 |
| 79 | 3 | 1 | 3 | 0 | 3 | 1 | | 3 | | 0 |
| 80 | 2 | 0 | 2 | 1 | 2 | 0 | | 2 | | 0 |
| 81 | 2 | 2 | 2 | 1 | 2 | 2 | | 2 | | 2 |
| 82 | 3 | 2 | 3 | 1 | 2 | 0 | | 3 | | 1 |
| 83 | 1 | 2 | 1 | 1 | 0 | 2 | | 1 | | 2 |
| 84 | 3 | 0 | 3 | 0 | 3 | 1 | | 3 | | 0 |
| 85 | 3 | 1 | 2 | 0 | 2 | 0 | | 2 | | 0 |
| 86 | 3 | 0 | 3 | 0 | 3 | 0 | | 3 | | 0 |
| 87 | 2 | 2 | 2 | 1 | 2 | 1 | | 2 | | 2 |
| 88 | 2 | 1 | 2 | 0 | 2 | 1 | | 2 | | 0 |
| 89 | 2 | 1 | 2 | 0 | 2 | 1 | | 2 | | 1 |
| 90 | 2 | 2 | 2 | 1 | 2 | 2 | | 2 | | 2 |
| 91 | 3 | 0 | 3 | 0 | 3 | 1 | | 3 | | 0 |
| 92 | 3 | 1 | 3 | 0 | 3 | 0 | | 3 | | 1 |
| 93 | 2 | 0 | 3 | 0 | 2 | 1 | | 2 | | 0 |
| 94 | 2 | 0 | 2 | 1 | 2 | 0 | | 2 | | 0 |
| 95 | 3 | 2 | 2 | 0 | 2 | 2 | | 2 | | 1 |
| 96 | 3 | 0 | 3 | 1 | 3 | 1 | | 3 | | 0 |
| 97 | 2 | 0 | 3 | 0 | 2 | 1 | | 2 | | 0 |
| 98 | 2 | 1 | 3 | 1 | 2 | 1 | | 2 | | 1 |
| 99 | 3 | 2 | 3 | 1 | 3 | 1 | | 3 | | 2 |
| 100 | 3 | 0 | 3 | 0 | 3 | 0 | | 3 | | 0 |
| 101 | 1 | 1 | 2 | 0 | 1 | 2 | | 1 | | 2 |
| 102 | 2 | 1 | 2 | 0 | 2 | 1 | | 2 | | 1 |
| 103 | 2 | 0 | 3 | 0 | 3 | 0 | | 3 | | 0 |
| 104 | 3 | 1 | 3 | 0 | 3 | 2 | | 3 | | 1 |
| 105 | 1 | 1 | 1 | 0 | 1 | 2 | | 1 | | 1 |
| 106 | 2 | 0 | 2 | 0 | 2 | 1 | | 2 | | 0 |
| 107 | 2 | 0 | 3 | 0 | 3 | 1 | | 3 | | 0 |
| 108 | 3 | 0 | 3 | 1 | 3 | 0 | | 3 | | 0 |
| 109 | 2 | 1 | 2 | 0 | 2 | 1 | | 2 | | 1 |
| 110 | 3 | 1 | 3 | 0 | 3 | 0 | | 3 | | 0 |
| 111 | 3 | 2 | 3 | 1 | 3 | 2 | | 3 | | 2 |
| 112 | 3 | 0 | 3 | 0 | 3 | 1 | | 3 | | 0 |
| 113 | 3 | 1 | 2 | 0 | 3 | 0 | | 2 | | 0 |
| 114 | 2 | 0 | 3 | 0 | 2 | 0 | | 3 | | 0 |
| 115 | 1 | 2 | 2 | 0 | 1 | 1 | | 2 | | 1 |
| 116 | 3 | 1 | 3 | 0 | 3 | 0 | | 3 | | 1 |
| 117 | 2 | 1 | 2 | 0 | 2 | 1 | | 2 | | 1 |
| 118 | 2 | 0 | 3 | 0 | 3 | 0 | | 3 | | 0 |
| 119 | 1 | 1 | 1 | 0 | 2 | 1 | | 2 | | 1 |
| 120 | 2 | 1 | 2 | 0 | 1 | 1 | | 2 | | 1 |
| 121 | 3 | 0 | 3 | 0 | 3 | 0 | | 3 | | 0 |
| 122 | 2 | 1 | 2 | 0 | 2 | 1 | | 2 | | 0 |
| 123 | 3 | 1 | 3 | 0 | 3 | 1 | | 3 | | 1 |
| 124 | 3 | 0 | 2 | 0 | 3 | 1 | | 2 | | 0 |
| 125 | 2 | 1 | 2 | 2 | 2 | 2 | | 2 | | 2 |
| 126 | 1 | 2 | 1 | 1 | 2 | 2 | | 2 | | 2 |
| 127 | 3 | 1 | 3 | 0 | 3 | 1 | | 3 | | 1 |
| 128 | 2 | 0 | 2 | 0 | 2 | 1 | | 2 | | 0 |
| 129 | 1 | 1 | 2 | 1 | 2 | 2 | | 1 | | 1 |
| 130 | 3 | 0 | 3 | 0 | 3 | 0 | | 3 | | 0 |
| 131 | 2 | 0 | 1 | 0 | 2 | 0 | | 1 | | 0 |
| 132 | 2 | 0 | 3 | 0 | 2 | 0 | | 2 | | 0 |
| 133 | 1 | 2 | 1 | 1 | 2 | 2 | | 2 | | 2 |
| 134 | 3 | 1 | 3 | 0 | 3 | 0 | | 3 | | 0 |
| 135 | 2 | 0 | 2 | 0 | 2 | 1 | | 2 | | 0 |
| 136 | 3 | 1 | 2 | 0 | 2 | 1 | | 2 | | 0 |
| 137 | 3 | 2 | 3 | 1 | 2 | 1 | | 2 | | 1 |
| 138 | 2 | 2 | 2 | 1 | 2 | 1 | | 2 | | 2 |
| 139 | 2 | 1 | 2 | 0 | 2 | 1 | | 2 | | 1 |
| 140 | 3 | 1 | 3 | 0 | 3 | 0 | | 3 | | 0 |
| 141 | 2 | 0 | 3 | 0 | 2 | 1 | | 2 | | 0 |
| 142 | 2 | 1 | 2 | 0 | 2 | 2 | | 2 | | 1 |
| 143 | 2 | 1 | 2 | 1 | 2 | 1 | | 2 | | 1 |
| 144 | 2 | 0 | 3 | 0 | 3 | 1 | | 2 | | 0 |
| 145 | 3 | 0 | 3 | 0 | 3 | 0 | | 3 | | 0 |
| 146 | 2 | 0 | 3 | 1 | 3 | 0 | | 3 | | 0 |
| 147 | 3 | 1 | 2 | 0 | 3 | 1 | | 2 | | 0 |
| 148 | 3 | 2 | 2 | 1 | 3 | 3 | | 3 | | 2 |
| 149 | 2 | 0 | 3 | 0 | 2 | 1 | | 2 | | 0 |
| 150 | 1 | 2 | 1 | 1 | 1 | 2 | | 1 | | 2 |
| 151 | 1 | 2 | 2 | 0 | 1 | 2 | | 1 | | 1 |
| 152 | 3 | 1 | 3 | 0 | 3 | 2 | | 3 | | 1 |
| 153 | 3 | 1 | 3 | 1 | 3 | 0 | | 3 | | 1 |
| 154 | 2 | 0 | 2 | 1 | 2 | 0 | | 2 | | 0 |
| 155 | 3 | 2 | 2 | 0 | 3 | 1 | | 2 | | 1 |
| 156 | 3 | 0 | 3 | 0 | 3 | 0 | | 3 | | 0 |
| 157 | 3 | 0 | 3 | 0 | 3 | 1 | | 3 | | 0 |
| 158 | 3 | 1 | 2 | 0 | 3 | 1 | | 2 | | 0 |
| 159 | 2 | 1 | 3 | 0 | 2 | 2 | | 2 | | 1 |
| 160 | 3 | 0 | 3 | 1 | 3 | 1 | | 2 | | 0 |
| 161 | 3 | 0 | 3 | 0 | 3 | 0 | | 3 | | 0 |
| 162 | 2 | 1 | 2 | 0 | 2 | 2 | | 2 | | 1 |
| 163 | 3 | 0 | 3 | 0 | 3 | 1 | | 3 | | 0 |
| 164 | 2 | 1 | 2 | 1 | 2 | 0 | | 2 | | 0 |
| 165 | 2 | 0 | 3 | 0 | 3 | 1 | | 2 | | 0 |
| 166 | 1 | 3 | 1 | 2 | 1 | 3 | | 1 | | 3 |
| 167 | 3 | 0 | 3 | 0 | 3 | 1 | | 3 | | 0 |
| 168 | 2 | 1 | 2 | 0 | 2 | 1 | | 2 | | 1 |
| 169 | 3 | 0 | 3 | 0 | 3 | 1 | | 3 | | 1 |
| 170 | 1 | 2 | 1 | 1 | 1 | 1 | | 1 | | 2 |
| 171 | 3 | 2 | 3 | 0 | 3 | 1 | | 3 | | 1 |
| 172 | 2 | 0 | 2 | 0 | 3 | 1 | | 2 | | 1 |
| 173 | 2 | 1 | 3 | 1 | 2 | 0 | | 2 | | 0 |
| 174 | 3 | 0 | 3 | 0 | 3 | 1 | | 3 | | 0 |
| 175 | 1 | 1 | 2 | 0 | 1 | 1 | | 2 | | 1 |
| 176 | 2 | 0 | 3 | 0 | 2 | 0 | | 2 | | 0 |
| 177 | 3 | 0 | 3 | 0 | 3 | 0 | | 3 | | 0 |
| 178 | 3 | 2 | 2 | 1 | 3 | 1 | | 2 | | 1 |
| 179 | 2 | 1 | 2 | 0 | 3 | 2 | | 1 | | 1 |
| 180 | 3 | 1 | 3 | 0 | 3 | 0 | | 3 | | 0 |
| 181 | 2 | 0 | 3 | 1 | 2 | 1 | | 2 | | 1 |
| 182 | 2 | 0 | 1 | 0 | 2 | 1 | | 2 | | 0 |
| 183 | 3 | 1 | 3 | 0 | 3 | 1 | | 3 | | 1 |
| 184 | 1 | 3 | 1 | 1 | 1 | 2 | | 1 | | 2 |
| 185 | 2 | 1 | 3 | 1 | 2 | 2 | | 2 | | 1 |
| 186 | 2 | 0 | 3 | 1 | 2 | 1 | | 2 | | 0 |
| 187 | 3 | 0 | 3 | 0 | 3 | 0 | | 3 | | 0 |
| 188 | 2 | 1 | 2 | 0 | 2 | 1 | | 2 | | 1 |
| 189 | 3 | 1 | 3 | 0 | 3 | 0 | | 3 | | 1 |
| 190 | 3 | 0 | 3 | 0 | 3 | 1 | | 3 | | 0 |
| 191 | 3 | 0 | 3 | 0 | 3 | 0 | | 3 | | 0 |
| 192 | 2 | 2 | 2 | 1 | 2 | 1 | | 2 | | 2 |
| 193 | 3 | 1 | 3 | 0 | 3 | 2 | | 3 | | 1 |
| 194 | 3 | 0 | 3 | 1 | 3 | 0 | | 3 | | 0 |
| 195 | 2 | 0 | 3 | 0 | 2 | 0 | | 3 | | 0 |
| 196 | 3 | 1 | 3 | 0 | 3 | 0 | | 3 | | 0 |
| 197 | 2 | 2 | 2 | 1 | 2 | 3 | | 2 | | 3 |
| 198 | 2 | 2 | 2 | 0 | 2 | 1 | | 2 | | 1 |
| 199 | 3 | 2 | 3 | 1 | 3 | 1 | | 3 | | 1 |
| 200 | 2 | 0 | 2 | 1 | 2 | 1 | | 2 | | 0 |
| 201 | 3 | 0 | 3 | 0 | 3 | 0 | | 3 | | 0 |
| 202 | 3 | 1 | 3 | 0 | 3 | 0 | | 3 | | 1 |
| 203 | 2 | 1 | 2 | 0 | 2 | 2 | | 2 | | 1 |
| 204 | 3 | 1 | 3 | 0 | 3 | 1 | | 3 | | 1 |
| 205 | 3 | 2 | 3 | 1 | 3 | 2 | | 3 | | 2 |
| 206 | 3 | 0 | 3 | 0 | 3 | 0 | | 3 | | 0 |
| 207 | 2 | 1 | 2 | 0 | 3 | 1 | | 2 | | 0 |
| 208 | 3 | 1 | 3 | 0 | 3 | 1 | | 3 | | 1 |
| 209 | 3 | 1 | 3 | 0 | 3 | 0 | | 3 | | 0 |
| 210 | 2 | 1 | 1 | 1 | 2 | 2 | | 2 | | 2 |
| 211 | 3 | 0 | 3 | 0 | 3 | 1 | | 3 | | 0 |
| 212 | 1 | 2 | 1 | 1 | 1 | 2 | | 1 | | 2 |
| 213 | 1 | 2 | 2 | 0 | 1 | 2 | | 2 | | 2 |
| 214 | 3 | 0 | 3 | 0 | 3 | 0 | | 3 | | 0 |
| 215 | 3 | 1 | 3 | 0 | 2 | 0 | | 2 | | 1 |
| 216 | 3 | 1 | 3 | 0 | 3 | 1 | | 3 | | 0 |
| 217 | 2 | 0 | 2 | 0 | 3 | 1 | | 2 | | 0 |
| 218 | 3 | 0 | 3 | 1 | 3 | 0 | | 3 | | 0 |
| 219 | 2 | 2 | 2 | 1 | 1 | 2 | | 2 | | 2 |
| 220 | 3 | 1 | 3 | 0 | 3 | 1 | | 3 | | 1 |
| 221 | 3 | 0 | 3 | 0 | 3 | 0 | | 3 | | 1 |
| 222 | 2 | 1 | 2 | 0 | 2 | 0 | | 2 | | 1 |
| 223 | 3 | 1 | 3 | 0 | 2 | 1 | | 2 | | 0 |
| 224 | 1 | 1 | 1 | 2 | 1 | 1 | | 2 | | 1 |
| 225 | 2 | 2 | 3 | 1 | 3 | 3 | | 3 | | 1 |
| 226 | 3 | 0 | 3 | 0 | 3 | 0 | | 3 | | 0 |
| 227 | 3 | 1 | 3 | 0 | 3 | 0 | | 3 | | 0 |
| 228 | 2 | 1 | 2 | 0 | 2 | 1 | | 2 | | 1 |
| 229 | 3 | 1 | 2 | 0 | 2 | 1 | | 2 | | 0 |
| 230 | 3 | 1 | 3 | 1 | 3 | 2 | | 3 | | 2 |
| 231 | 1 | 2 | 2 | 0 | 1 | 1 | | 1 | | 1 |
| 232 | 3 | 0 | 3 | 0 | 3 | 1 | | 3 | | 0 |
